# Chemical characteristics of atmospheric precipitation and their effects on microbial diversity in Baotou, China

**DOI:** 10.3389/fmicb.2025.1680819

**Published:** 2025-10-13

**Authors:** Lili Wang, Yuping Yang, Li Gao, Zhichun Gao

**Affiliations:** ^1^College of Ecology and Environment, Baotou Teacher’s College, Baotou, China; ^2^Baotou Branch, Inner Mongolia Environmental Monitoring Center, Baotou, China

**Keywords:** atmospheric precipitation, chemical composition, microbial diversity, environmental factors, community assembly, functional prediction

## Abstract

**Introduction:**

Existing studies on the coupling of atmospheric precipitation chemistry and microbial communities have long focused on humid regions, while overlooking the unique “sand-dust-agriculture-industry” compound pollution in arid/semi-arid industrial cities—creating a critical knowledge gap. This study aimed to systematically explore the interactive mechanisms, community assembly processes, and ecological/health implications of precipitation-associated microbes in Baotou, a typical heavy-industry hub in northern China.

**Methods:**

Precipitation samples were collected from May to August 2023 and analyzed for chemical ions (Ca^2+^, NH_₄_^+^, SO_₄_^2−^, etc.) and microbial communities via ion chromatography and high-throughput sequencing of 16S/18S rRNA genes. Community assembly processes were assessed using null model analyses (NST, iCAMP), and microbial functions were predicted via FAPROTAX and FUNGuild.

**Results:**

Baotou’s precipitation exhibited a neutral pH (7.04 ± 0.14) but abnormally high ion loading (795.09 ± 94.68 μeq·L^−1^), dominated by Ca^2+^, NH_₄_^+^, and SO_₄_^2−^, reflecting mixed dust, agricultural, and industrial sources. Microbial community assembly was predominantly stochastic (drift + dispersal limitation >79% in bacteria and >86% in fungi), with fungi showing significantly broader niche width and overlap than bacteria (*p*<0.05). Functionally, bacteria were primarily involved in carbon and nitrogen cycling, whereas fungi displayed balanced saprotrophic (48.03%) and pathogenic (48.24%) traits. Fungal pathogens (e.g., *Cladosporium, Alternaria*) were significantly more abundant than bacterial pathogens, forming a distinct “fungal-dominated” health risk profile.

**Discussion:**

The dominance of stochastic assembly and the functional divergence between bacteria and fungi underscore unique microbial adaptive strategies under compound pollution stress in arid industrial atmospheres. The high abundance of allergenic fungal pathogens highlights significant public health risks, especially during warm seasons. This study provides a novel framework for understanding precipitation chemistry-microbe interactions in arid industrial environments, offering critical insights for regional air quality management and health risk assessment.

## 1 Introduction

Atmospheric precipitation serves as a crucial scavenger of airborne pollutants and microorganisms, playing a pivotal role in regulating atmospheric chemistry and microbial dispersal. Numerous studies in humid regions—where frequent rainfall facilitates deposition—have established clear relationships between precipitation characteristics (e.g., pH, ion concentrations) and microbial dynamics, highlighting how environmental factors shape microbial community structure and function (Hiraoka et al., 2017; Bowers et al., 2013; Górka et al., 2024; Si and Li, 2024). In contrast, arid and semi-arid industrial cities, which experience a unique combination of infrequent precipitation, frequent dust events, and intense anthropogenic emissions, remain significantly understudied. The chemical and biological characteristics of atmospheric precipitation are key indicators of regional environmental quality and are intimately linked to ecological security and public health (Malnik et al., 2024). This knowledge gap is critical because the distinct environmental conditions—such as high ion loading and mixed pollution sources—likely foster unique microbial ecological patterns that cannot be extrapolated from studies in humid regions.

As a quintessential heavy industrial hub in China’s arid/semi-arid region, Baotou’s unique atmospheric environment stems from its steel manufacturing, coal chemical industries, and rare earth metallurgy, compounded by persistent dust storms. This creates a dual pollution paradigm: coal-dominated energy consumption emits substantial SO₂ and NOₓ (Zhen, 2012), while frequent dust events elevate particulate matter and crustal ions (e.g., Ca^2+^) (Zhou et al., 2018). Consequently, precipitation exhibits elevated soluble ions (Ca^2+^, SO₄^2−^, etc.), creating a unique medium that acts as both a sink for pollutants and a potential growth substrate for microbes. While nitrogen/sulfur compounds may provide nutrients, osmotic stress from high ion concentrations could inhibit microbial survival (Bowers et al., 2013), making these chemicals pivotal mediators between atmospheric chemistry and microbial ecology. Currently, research on atmospheric precipitation in Baotou has mainly focused on chemical characteristics (Gao et al., 2024), with a lack of systematic analysis of microbial communities and their environmental interactions. Although studies elsewhere suggest that precipitation microbial communities are highly sensitive to environmental regulation (Lu et al., 2016), and that reduced precipitation may alter microbial networks in arid regions (Wang et al., 2023), such effects remain unelucidated in arid/semi-arid industrial cities.

To address this research gap, we conducted a comprehensive study during the peak precipitation period (May–August 2023) in Baotou, combining high-throughput sequencing with chemical and meteorological analyses. Our research aims to: (1) characterize the pollution sources and ionic composition of precipitation in this industrial arid region; (2) elucidate the diversity and structure of microbial communities in precipitation; (3) identify key environmental drivers shaping microbial assembly mechanisms; and (4) assess the ecological and health risks associated with microbial functional traits. This study provides the first comprehensive investigation of precipitation microbiology in an arid industrial city, offering novel insights into the coupling mechanism of precipitation chemistry and microbial communities. The findings aim to support atmospheric pollution control and ecological/health risk assessment in arid/semi-arid industrial regions like Baotou.

## 2 Materials and methods

### 2.1 Precipitation sample collection

Precipitation samples were collected at a provincial-level air quality monitoring station in Baotou City (Environmental Monitoring Station: 40.6181°N, 109.9169°E) during the peak precipitation period from May to August 2023. A total of 12 precipitation samples were obtained (JS05-1 ~ 3 in May, JS06-1 ~ 3 in June, JS07-1 ~ 3 in July, and JS08-1 ~ 3 in August). Sampling was conducted in strict compliance with the Chinese national standard “Technical Specifications for Acid Deposition Monitoring (HJ/T 165–2004)” (State Environmental Protection Administration of the People’s Republic of China, 2004). We employed a 24-h cumulative sampling method using polyethylene containers (40 cm diameter × 20 cm height) to collect complete precipitation events. For continuous precipitation, cumulative samples were collected from 9:00 to 9:00 a.m. the following day. Immediately after collection, samples were transported to the laboratory. After homogenization, each sample was divided into two aliquots: one aliquot was directly used for chemical characteristic analysis, and the other was filtered through a 0.22 μm microporous membrane. The filtered membranes were stored in a −80 °C for subsequent microbial analysis.

### 2.2 Chemical and meteorological analysis

Chemical analyses of precipitation samples were conducted following standardized national methods: Conductivity was measured using a Leici DDSJ-308A conductivity meter according to GB 13580.3–1992 (State Environmental Protection Administration of the People’s Republic of China, 1992a). PH was determined with a Mettler Toledo S210 pH meter following GB 13580.4–1992 (State Environmental Protection Administration of the People’s Republic of China, 1992b). Anions (F^−^, Cl^−^, NO₃^−^, SO₄^2−^) were analyzed using a Dionex ICS-1100 ion chromatograph. Samples were filtered through a 0.45-μm membrane prior to analysis following GB 13580.5–1992 (State Environmental Protection Administration of the People’s Republic of China, 1992c). Cations (K^+^, Na^+^, Ca^2+^, Mg^2+^) were analyzed using a Dionex ICS-1000 ion chromatograph according to HJ 1005–2018 (Ministry of Ecology and Environment of the People's Republic of China, 2018). NH₄^+^ Concentration was determined by the Nessler reagent photometric method following GB 13580.11–1992 (State Environmental Protection Administration of the People’s Republic of China, 1992d). Simultaneously, hourly concentrations of SO₂, NO₂, and O₃ were obtained from the automated air quality monitoring station co-located with the precipitation sampling site. Concurrent atmospheric data (SO₂, NO₂, O₃) were obtained from the sampling site’s automatic air quality monitoring station. Meteorological parameters including temperature (TEMP), relative humidity (RH), and wind speed (WS) were acquired through the Air Quality Online Monitoring Analysis Platform.^1^ All reported pH, conductivity, and ion concentrations were calculated as precipitation-weighted averages using Equation 1 (Li et al., 2023):


(1) $$C=\sum_{i=1}^{n}C_{i}\times P_{i}/\sum_{i=1}^{n}P_{i}$$

where *C*_*i*​_ represents the pH, conductivity (μS·cm^−1^), or ion concentration (μeq·L^−1^) of each precipitation event, and *P*_*i*​_ denotes the corresponding precipitation amount (mm).

### 2.3 16S/18S rRNA amplicon sequencing

Precipitation samples were filtered through 0.22 μm membranes, followed by microbial DNA extraction using the DNeasy PowerSoil Pro Kit (QIAGEN). The quality and quantity of the extracted DNA were assessed by electrophoresis on a 1.8% agarose gel, and the concentration and purity were measured using a NanoDrop 2000 UV–Vis spectrophotometer (Thermo Scientific, Wilmington, United States). The V3–V4 hypervariable regions of the bacterial 16S rRNA gene were amplified with the primers 338F (5′-ACTCCTACGGGAGGCAGCA-3′) and 806R (5′-GGACTACHVGGGTWTCTAAT-3′) (Takahashi et al., 2014). The ITS1 region of the fungal 18S rRNA gene was amplified using primers ITS1F (5′-CTTGGTCATTTAGAGGAAGTAA-3′) (Gardes and Bruns, 1993) and ITS2 (5′-GCTGCGTTCTTCATCGATGC-3′) (White et al., 1990). Both forward and reverse primers were tailed with sample-specific Illumina index sequences to enable multiplexed deep sequencing. Each sample was amplified in three independent PCR replicates, which were then pooled. The amplified products were purified with the Omega DNA purification kit (Omega Inc., Norcross, GA, United States) and quantified using a Qsep-400 system (BiOptic, Inc., New Taipei City, Taiwan, China). The amplicon library was subjected to paired-end sequencing (2 × 250 bp) on an Illumina NovaSeq 6,000 platform (Beijing Biomarker Technologies Co., Ltd., Beijing, China).

### 2.4 Statistical analysis

Raw sequencing reads were quality-filtered using Trimmomatic (v0.33), followed by primer removal with Cutadapt (v1.9.1). Paired-end reads were merged and chimeras were removed using USEARCH (v10). High-quality sequences were clustered into operational taxonomic units (OTUs) at 97% similarity and taxonomically annotated against the SILVA 138 database. To ensure the use of updated taxonomic nomenclature, all bacterial OTUs were subsequently re-annotated using the SILVA release 138.2 reference database (the latest stable version, released July 11, 2024). To further validate taxonomic accuracy, all prokaryotic OTU annotations were cross-referenced with the List of Prokaryotic Names with Standing in Nomenclature (LPSN)^2^. To account for sequencing depth variation, all samples were rarefied to the minimum read count (47,682 reads for bacteria; 34,594 reads for fungi).

Alpha diversity was assessed using the Simpson Index, Shannon Index, ACE (Abundance-based Coverage Estimator) Index, and Chao1 Index, which collectively characterize microbial species richness, evenness, and potential rare taxa richness in atmospheric precipitation samples. Beta diversity was analyzed via principal coordinates analysis (PCoA) based on the Binary-Jaccard distance matrix—this approach quantifies turnover in microbial community composition by integrating taxonomic presence/absence and relative abundance, a robust method for characterizing compositional differences in atmospheric precipitation studies (Nageen et al., 2023; Wang et al., 2023). Significance testing of differences in community composition (reflected by PCoA clustering) between groups was performed using ANOSIM with 999 permutations. The relationships between environmental factors and microbial communities were analyzed using canonical correspondence analysis (CCA). Spearman correlation coefficients between taxa and environmental variables were calculated (*p <* 0.05, *p <* 0.01) and visualized via heatmaps. Differential abundance analysis was conducted using the linear discriminant analysis effect size (LEfSe) method (LDA score >2.0, *p <* 0.05). Functional predictions were performed using FAPROTAX for bacterial metabolic functions and FunGuild for fungal ecological guilds. The aforementioned analyses were conducted on the BMKCloud platform.^3^

Community assembly mechanisms were investigated through two approaches: (1) Quantifying the contribution of stochastic processes using the normalized stochasticity ratio (NST) based on the Bray-Curtis distance matrix and the “PF” null model algorithm (Ning et al., 2019); (2) Quantifying the relative importance of ecological processes using the iCAMP method, where βNRI >1.96 indicates heterogeneous selection (HeS), βNRI <−1.96 indicates homogeneous selection (HoS), |βNRI| ≤ 1.96 and RC < −0.95 indicates homogenizing dispersal (HD), |βNRI| ≤ 1.96 and RC > 0.95 indicates dispersal limitation (DL), and |βNRI| ≤ 1.96 and |RC| ≤ 0.95 indicates drift (DR) (Ning et al., 2019). Additionally, niche breadth and niche overlap indices of the top 30 dominant OTUs were calculated using the “niche.width” and “niche.overlap” functions (method = “levins”) in the spaa package to evaluate environmental adaptability and interspecific competition. All statistical analyses were performed in R 4.2.0.

## 3 Results

### 3.1 Chemical characteristics of atmospheric precipitation

Chemical analysis of atmospheric precipitation in Baotou from May to August showed that the precipitation-weighted pH ranged from 6.82 to 7.16 (mean±SD, 7.04 ± 0.14), indicating neutral characteristics with no acidification trend (Table 1). The precipitation-weighted conductivity varied between 39.74 and 88.25 μS·cm^−1^ (mean±SD, 65.38 ± 20.63μS·cm^−1^), significantly higher than that at Waliguan, a background site for atmospheric precipitation in China (15.08 μS·cm^−1^) (Zhang et al., 2014). This reflects poor precipitation cleanliness in the study area, with high levels of pollutants such as water-soluble ions in precipitation. The precipitation-weighted total ion concentration ranged from 695.55 to 912.56 μeq·L^−1^ (mean±SD, 795.09 ± 94.68μeq·L^−1^), which is higher than that in most cities in China (Xu et al., 2020), consistent with the high conductivity measurements. Ionic composition followed the order: Ca^2+^ > NH₄^+^ > SO₄^2−^ > Na^+^ > Cl^−^ > Mg^2+^ > NO₃^−^ > F^−^ > K^+^. Among these, Ca^2+^, NH₄^+^, and SO₄^2−^ accounted for 66.49% of the total ion concentration, making them the dominant ions during the monitoring period. Ca^2+^ was the most abundant cation, contributing 27.56% to the total ions and representing a major pollutant in precipitation. Previous studies have identified Ca^2+^ as an indicator of soil dust sources; in Baotou, frequent sand-dust events in May and June result in substantial Ca^2+^ input from airborne dust, consistent with the region’s sand-dust pollution characteristics (Gao et al., 2024). NH₄^+^ was the second most abundant cation, accounting for 21.57% of total ions—higher than the typical <10% reported in previous studies. This elevation may be linked to agricultural activities in the surrounding areas, such as spring fertilization (Hůnová et al., 2022). SO₄^2−^ was the dominant anion, comprising 17.35% of total ions and acting as the primary acidic ion in precipitation. Its high concentration is primarily attributed to sulfate formation via atmospheric oxidation of sulfur dioxide (SO₂) from coal combustion, aligning with Baotou’s coal-dominated energy consumption structure.

**Table 1:** Chemical characteristics of atmospheric precipitation in Baotou (May–August 2023).

Month	Precipitation/mm	pH	Conductivity /(μS·cm^−1^)	Ions concentration/(μeq·L^−1^)									Total ion concentration/(μeq·L^−1^)
				SO_4_^2−^	NO_3_^−^	NH_4_^+^	F^−^	Cl^−^	K^+^	Na^+^	Ca^2+^	Mg^2+^	
May	8.00	7.16	72.10	119.52	30.66	108.00	18.30	59.67	27.46	74.15	212.49	45.31	695.55
June	4.45	7.11	88.25	106.06	31.81	227.48	13.92	69.77	14.52	98.50	297.47	53.03	912.56
July	11.08	6.82	39.74	92.52	37.46	167.22	17.85	69.63	11.72	99.79	173.64	50.21	720.04
August	6.77	7.06	61.42	233.78	45.49	183.24	40.41	45.27	13.09	47.44	193.04	50.45	852.20
Mean±SD	7.58 ± 2.76	7.04 ± 0.14	65.38 ± 20.63	137.97 ± 66.98	36.36 ± 6.19	171.48 ± 47.72	22.62 ± 11.90	61.08 ± 10.60	16.70 ± 6.69	79.97 ± 23.42	219.16 ± 50.25	49.75 ± 3.40	795.09 ± 94.68

**Figure 1:**
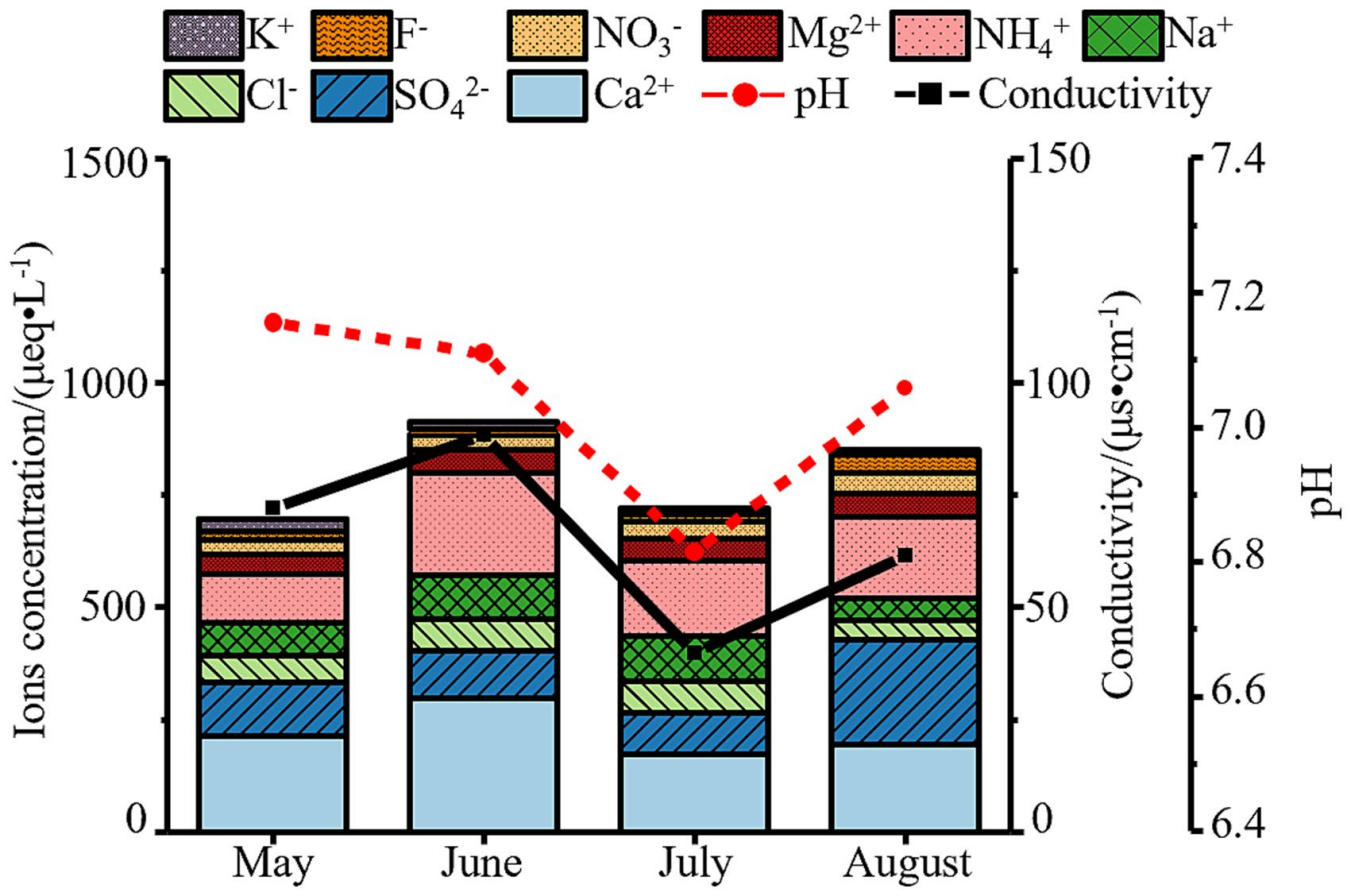
Variations in pH, conductivity and ion concentrations of atmospheric precipitation in Baotou (May–August 2023).

Overall, ion concentrations in atmospheric precipitation exhibited significant monthly variations (Figure 1), with a peak in June and a decline in July—consistent with trends in conductivity but opposite to precipitation amounts. During the monitoring period, July recorded the highest precipitation (11.08 mm), and intense rainfall diluted atmospheric ions: concentrations of SO₄^2−^ (92.52 μeq/L) and Ca^2+^ (173.64 μeq/L) dropped to their lowest levels, leading to a decrease in total ion concentration. In contrast, June had minimal precipitation (4.55 mm), weakening dilution effects. Combined with frequent sand-dust events (Ca^2+^ peaking at 297.47 μeq/L) and nitrogen fertilizer volatilization during active agricultural periods (NH₄^+^ peaking at 227.48 μeq/L), total ion concentration increased in June. Notably, active photochemical reactions in August led to the highest SO₄^2−^ concentration (233.78 μeq/L) during the monitoring period, reflecting seasonal declines in coal-fired desulfurization efficiency in Baotou.

### 3.2 Microbial diversity in atmospheric precipitation

High-throughput sequencing yielded 959,808 raw bacterial sequences and 887,646 raw fungal sequences. After quality filtering, denoising, assembly, and chimera removal, 642,089 bacterial and 717,049 fungal high-quality sequences were retained. Based on a 97% sequence similarity threshold, these sequences were clustered into 6,153 bacterial operational taxonomic units (OTUs) and 3,303 fungal OTUs. To account for differences in sequencing depth, all samples were normalized to the minimum read counts (47,682 reads for bacteria; 34,594 reads for fungi), resulting in 6,144 bacterial OTUs and 3,211 fungal OTUs for subsequent analyses. The bacterial OTUs were classified into 35 phyla, 79 classes, 217 orders, 412 families, and 856 genera, while the fungal OTUs were assigned to 12 phyla, 37 classes, 95 orders, 238 families, and 521 genera.

**Figure 2:**
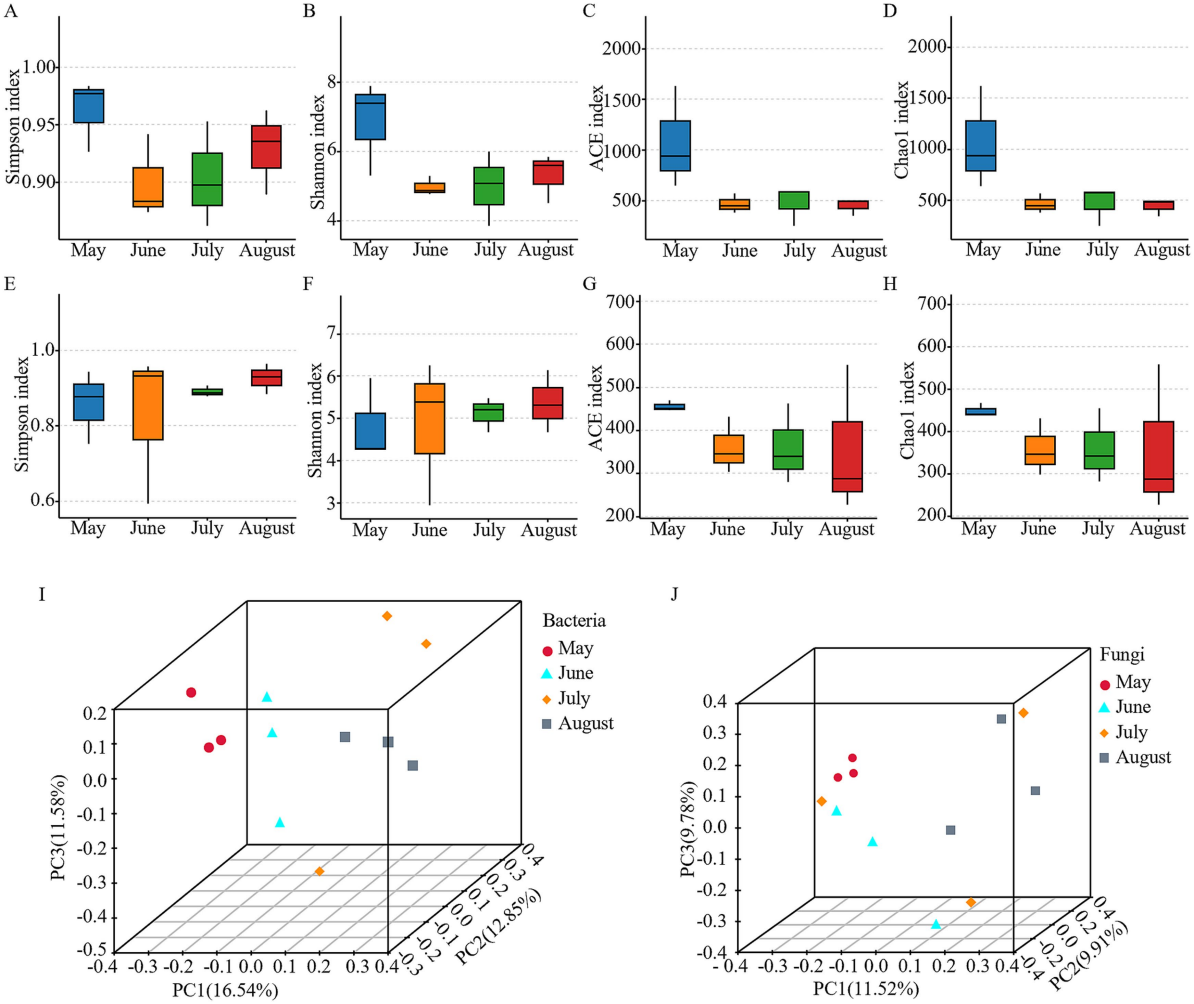
Microbial diversity analysis in atmospheric precipitation samples. **(A)** Bacterial Simpson indices. **(B)** Bacterial Shannon diversity. **(C)** Bacterial abundance-based coverage estimator (ACE) index. **(D)** Bacterial chao1 diversity. **(E)** Fungal Simpson indices. **(F)** Fungal Shannon diversity. **(G)** Fungal abundance-based coverage estimator (ACE) index. **(H)** Fungal chao1 diversity. **(I)** Bacterial *β*-diversity PCoA (principal coordinate analysis). **(J)** fungal β-diversity PCoA.

Rarefaction curves indicated that OTU counts for both bacteria and fungi reached a plateau with increasing sequencing depth, confirming sufficient sequencing coverage to accurately represent the majority of microbial diversity in the samples (Supplementary Figure 1). Alpha diversity of the bacterial and fungal communities was assessed using four indices: the Simpson and Shannon indices (reflecting community evenness and diversity) and the ACE and Chao1 indices (estimating species richness). The ACE and Chao1 indices indicated high species richness in both communities (Figures 2C,D,G,H). Bacterial communities demonstrated uniformly high diversity and evenness, as evidenced by consistently high Simpson and Shannon indices (Figures 2A,B). In contrast, fungal communities exhibited substantial variation in diversity and evenness among samples (Figures 2E,F). However, statistical analysis (Kruskal-Wallis test) revealed no significant monthly variations in any of the four alpha diversity indices for either bacteria or fungi (*p* > 0.05; Figures 2A–H), suggesting that the within-month diversity remained relatively stable over time. In contrast to the stable alpha diversity, the beta diversity analysis revealed significant temporal dynamics. Principal coordinates analysis (PCoA) revealed significant monthly variations in bacterial and fungal community compositions, as evidenced by clear clustering patterns (except for the dispersed July samples) and distinct separations between months (Figures 2I,J). ANOSIM further confirmed significant compositional differences across months for both bacteria (*R* = 0.586, *p* = 0.002) and fungi (*R* = 0.448, *p* = 0.008).

### 3.3 Microbial community composition in atmospheric precipitation

**Figure 3:**
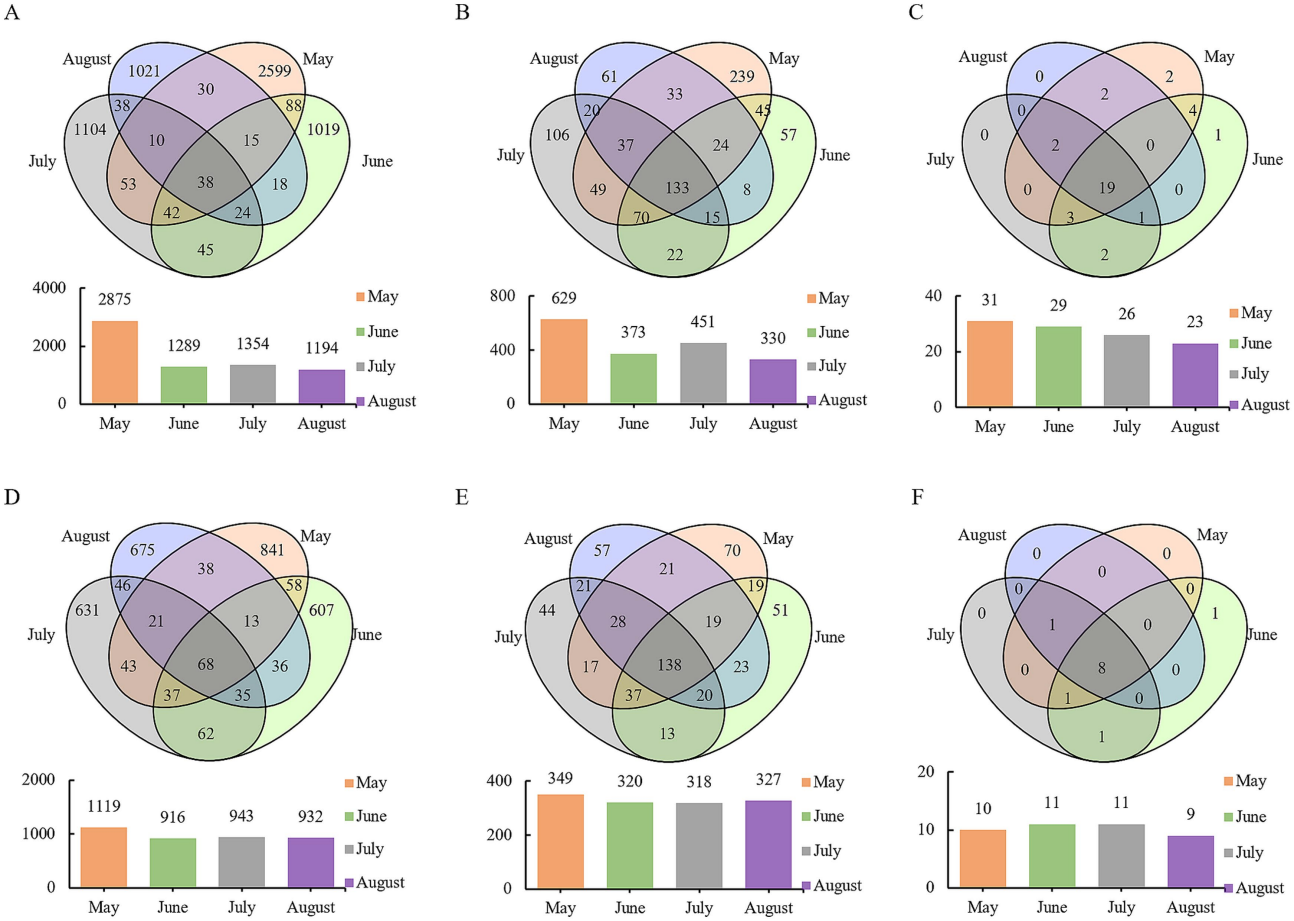
Venn analysis of microbial communities in atmospheric precipitation. **(A)** Bacterial OTUs. **(B)** Bacterial genera. **(C)** Bacterial phyla. **(D)** Fungal OTUs. **(E)** Fungal genera. **(F)** Fungal phyla.

Venn analysis revealed that among the 6,144 bacterial OTUs, only 38 OTUs were shared across all months, accounting for less than 1% of the total, with the highest number of OTUs observed in May (Figure 3A). For fungal OTUs (3,211 in total), 68 OTUs were shared across months, representing 2% of the total, and similarly, the highest number of OTUs was recorded in May (Figure 3D). Further analysis of phylum- and genus-level variations showed that 19 bacterial phyla, 133 bacterial genera, eight fungal phyla, and 138 fungal genera were common to all months. Additionally, each month contained unique genera, with the greatest number observed in May—consistent with the findings at the OTU level (Figure 3). Collectively, these patterns reveal shared microbial taxa across months, reflecting baseline commonalities in community composition. Each month, however, harbors distinct assemblages, underscoring the temporal diversity and specificity of precipitation-associated microbial communities.

**Figure 4:**
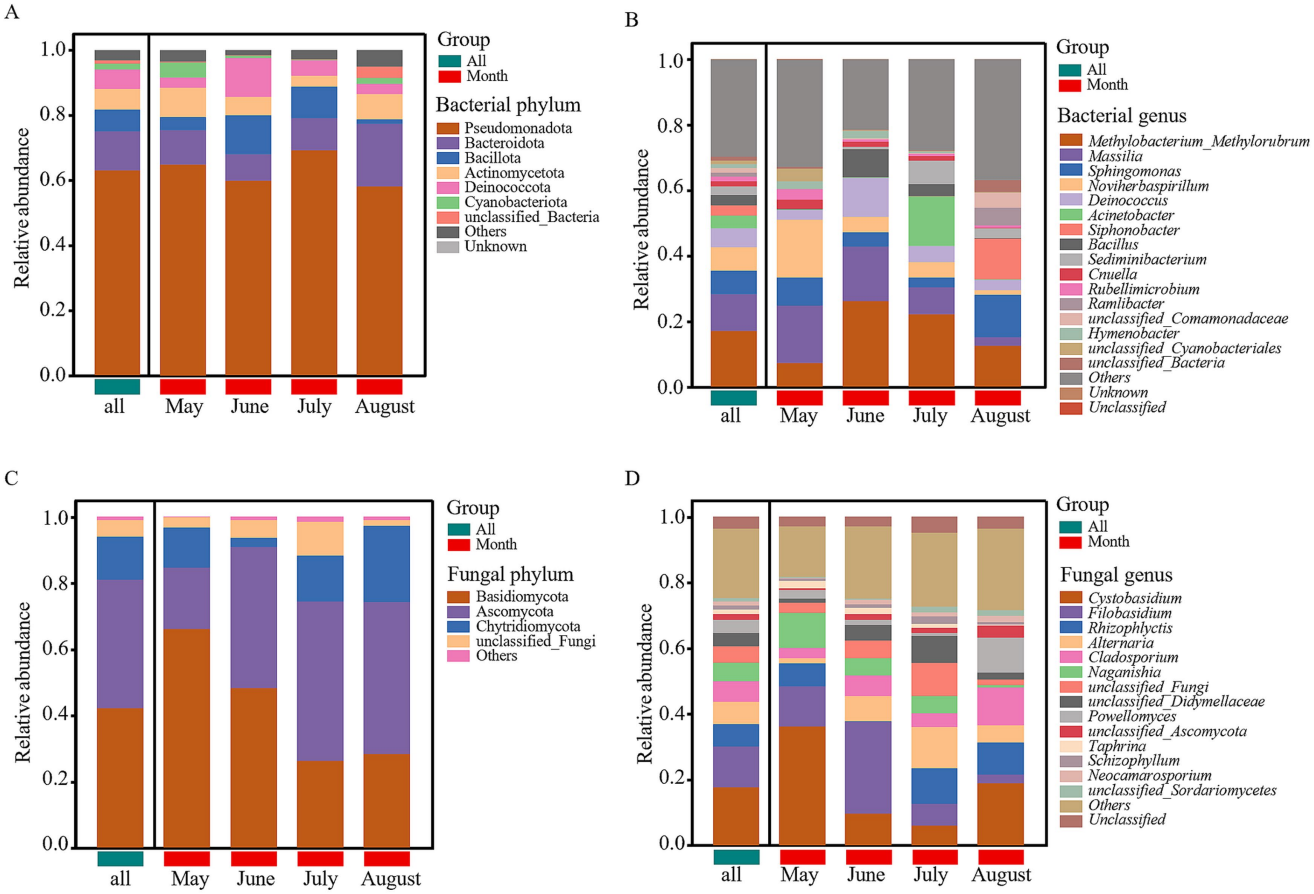
Relative abundance of microbial communities in atmospheric precipitation. **(A)** Bacterial community composition at the phylum level. **(B)** Bacterial community composition at the genus level. **(C)** Fungal community composition at the phylum level. **(D)** Fungal community composition at the genus level. Only phyla/genera with a relative abundance >1% are shown; low-abundance taxa are consolidated into “Others.”

**Figure 5:**
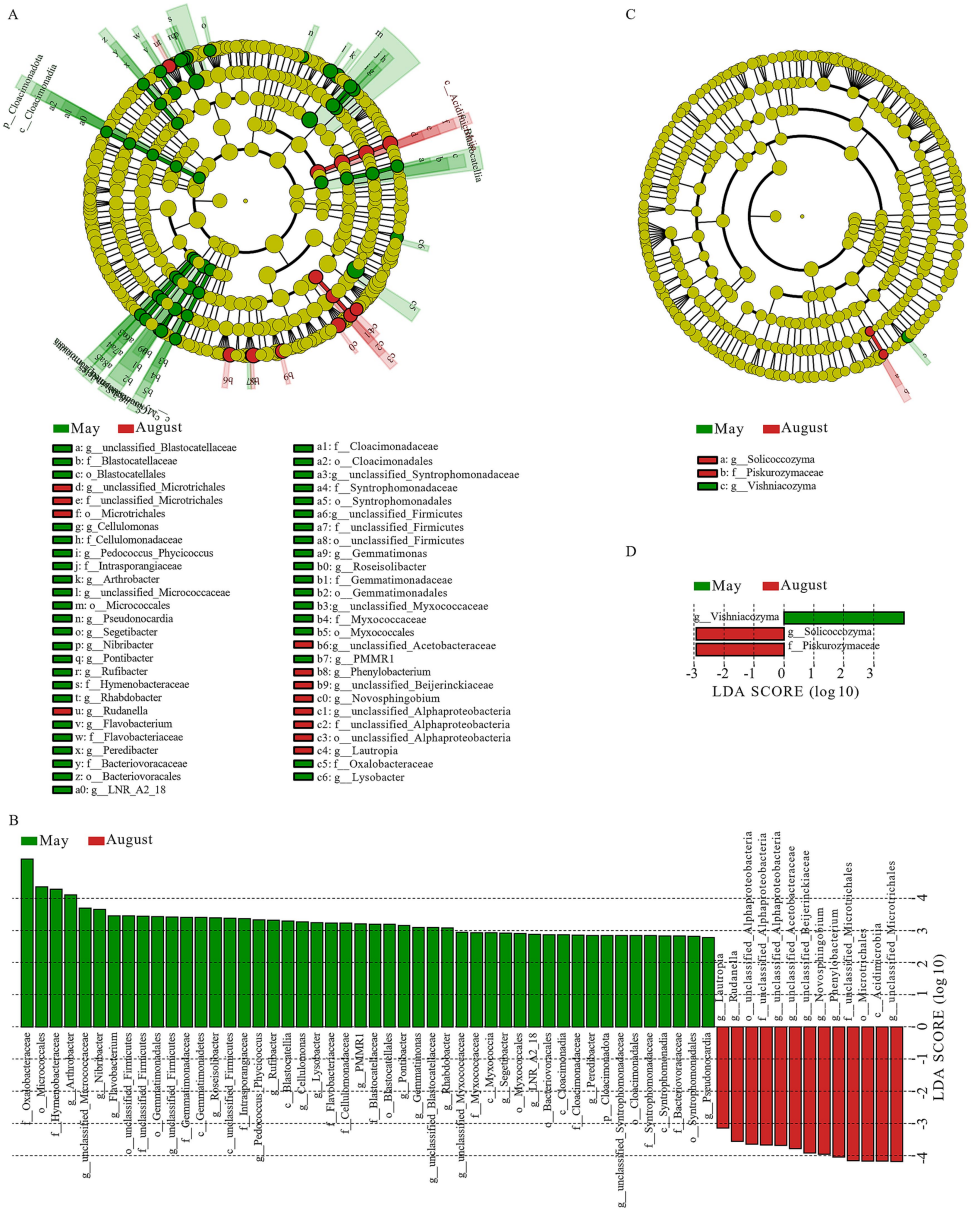
LEfSe analysis of bacterial and fungal community groups in atmospheric precipitation. **(A)** Bacterial cladogram. **(B)** Bacterial LDA bar chart (LDA > 2.0, *p* < 0.05). **(C)** Fungal cladogram. **(D)** Fungal LDA bar chart (LDA > 2.0, *p* < 0.05).

After filtering unannotated and ambiguous taxa, and consolidating low-abundance phyla (<1%) into “others,” we identified six dominant bacterial phyla (Pseudomonadota, Bacteroidota, Bacillota, Actinomycetota, Deinococcota, and Cyanobacteriota) and three dominant fungal phyla (Basidiomycota, Ascomycota, and Chytridiomycota) (Figures 4A,C). Among these, Pseudomonadota, Bacteroidota, Bacillota, and Actinomycetota were the most dominant bacterial phyla (relative abundance >6%), while Basidiomycota and Ascomycota were the most dominant fungal phyla (relative abundance >15%). Within the dominant phylum Pseudomonadota, Alphaproteobacteria (mean relative abundance: 33.51%) and Gammaproteobacteria (29.36%) were the two predominant classes, collectively accounting for over 95% of this phylum’s abundance and exhibiting dynamic shifts across months (Supplementary Table 1). At the genus level (after similar filtering), 16 major bacterial and 14 fungal genera were identified (Figures 4B,D). Among these, Methylobacterium_Methylorubrum, Massilia, Sphingomonas, and Noviherbaspirillum were the most dominant bacterial genera (relative abundance >6%), while Cystobasidium, Filobasidium, Rhizophlyctis, Alternaria, and Cladosporium were the most dominant fungal genera (relative abundance >6%). LEfSe analysis (LDA score >2, *p* < 0.05) performed at higher taxonomic ranks revealed clear monthly shifts in the microbial community structure, providing a broader ecological overview (Figure 5). The microbial community in May was significantly enriched with a variety of taxa, including the phylum Cloacimonadota, classes such as Syntrophomonadia, Blastocatellia, and Myxococcia (six classes total), and families including Syntrophomonadaceae, Myxococcaceae, and Blastocatellaceae (12 families total). In contrast, the August community was less diverse and dominated by a different set of taxa, primarily the class Acidimicrobiia and the families unclassified_Microtrichales and unclassified_Alphaproteobacteria (two families total). For fungi, the most significant differences were at the genus level, with Vishniacozyma enriched in May and Solicoccozyma enriched in August.

### 3.4 Environmental drivers of microbial communities

Environmental factors significantly influenced microbial community structures in atmospheric precipitation. We employed canonical correspondence analysis (CCA) analyses to examine the relationships between key environmental parameters (meteorological factors: PRCP, TEMP, RH, WS, NO_2_, SO_2_, O_3_; precipitation physicochemical factors: pH, SO₄^2−^, NO₃^−^, NH₄^+^, Cl^−^, K^+^, Na^+^, Ca^2+^, Mg^2+^) and the top 20 dominant bacterial and fungal genera in Baotou’s precipitation.

**Figure 6:**
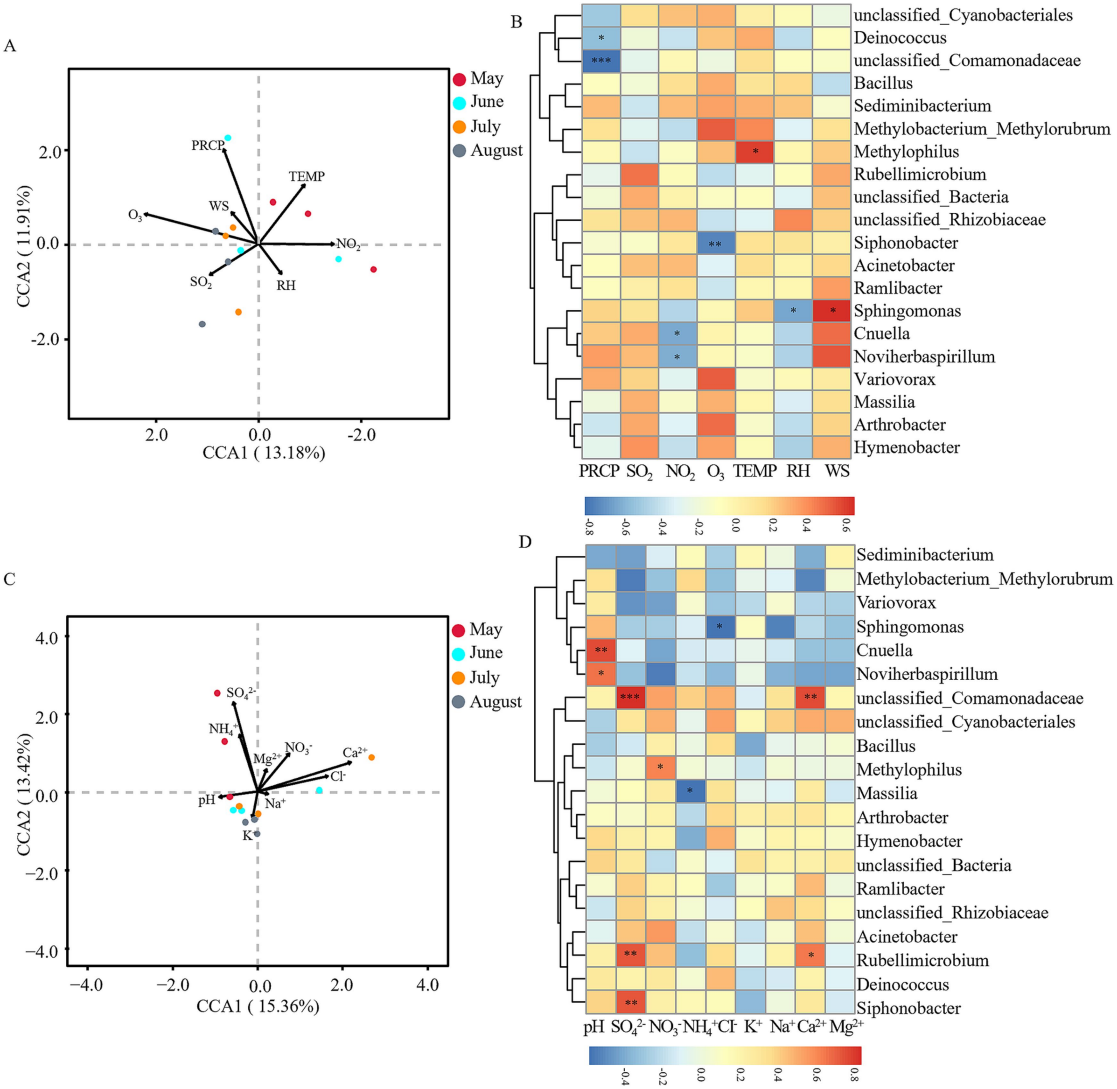
Correlation analysis between environmental factors and bacterial communities. **(A)** Canonical correspondence analysis (CCA) of meteorological factors vs. bacterial communities at the OTU level. **(B)** Spearman correlation analysis of meteorological factors vs. dominant bacterial genera at the genus level. **(C)** CCA of precipitation physicochemical factors vs. bacterial communities at the OTU level. **(D)** Spearman correlation analysis of precipitation physicochemical factors vs. dominant bacterial genera at the genus level. Arrows indicate environmental variables, circles represent sample points (color-coded by month). *p < 0.05, **p < 0.01, ***p < 0.001. Meteorological factors comprised precipitation (PRCP), temperature (TEMP), relative humidity (RH), wind speed (WS), and gaseous pollutants (SO₂, NO₂, O₃). Precipitation physicochemical properties included pH and major ions (SO₄^2−^, NO₃^−^, NH₄^+^, Cl^−^, K^+^, Na^+^, Ca^2+^, Mg^2+^). Note: Color scales are automatically generated and optimized for each individual panel to best represent the structure within each dataset; therefore, direct visual comparison of color intensity between panels is not advised.

For bacterial communities, CCA indicated that meteorological factors accounted for 25.09% of the community variation, with O₃ (*R*^2^ = 0.581, *p* = 0.024) identified as the most significant driver (Figure 6A). Spearman correlation heatmaps revealed distinct associations between specific bacterial taxa and meteorological factors: unclassified_Comamonadaceae (*r* = −0.830, *p* < 0.001) and Deinococcus (*r* = −0.578, *p* < 0.05) showed significant negative correlations with PRCP; Cnuella (*r* = −0.650, *p* < 0.05) and Noviherbaspirillum (*r* = −0.636, *p* < 0.05) were negatively correlated with NO₂; Siphonobacter (*r* = −0.764, *p* < 0.01) was negatively correlated with O₃; Methylophilus was positively correlated with TEMP (*r* = 0.594, *p* < 0.05); Sphingomonas was negatively correlated with RH (*r* = −0.657, *p* < 0.05) but positively correlated with WS (*r* = 0.643, *p* < 0.05) (Figure 6B). Physicochemical factors explainedc of the variation, although no single factor reached statistical significance (Figure 6C). The Spearman heatmap highlighted several specific correlations: Cnuella (*r* = 0.748, *p* < 0.01) and Noviherbaspirillum (*r* = 0.657, *p* < 0.05) were positively correlated with precipitation pH; unclassified_Comamonadaceae (*r* = 0.832, *p* < 0.001), Rubellimicrobium (*r* = 0.726, *p* < 0.01), and Siphonobacter (*r* = 0.720, *p* < 0.01) were positively correlated with SO₄^2−^; Methylophilus (*r* = 0.616, *p* < 0.05) was positively correlated with NO₃^−^; Massilia (*r* = −0.580, *p* < 0.05) was negatively correlated with NH₄^+^; Sphingomonas (*r* = −0.581, *p* < 0.05) was negatively correlated with Cl^−^; unclassified_Comamonadaceae (*r* = 0.748, *p* < 0.01) and Rubellimicrobium (*r* = 0.643, *p* < 0.05) were positively correlated with Ca^2+^ (Figure 6D).

**Figure 7:**
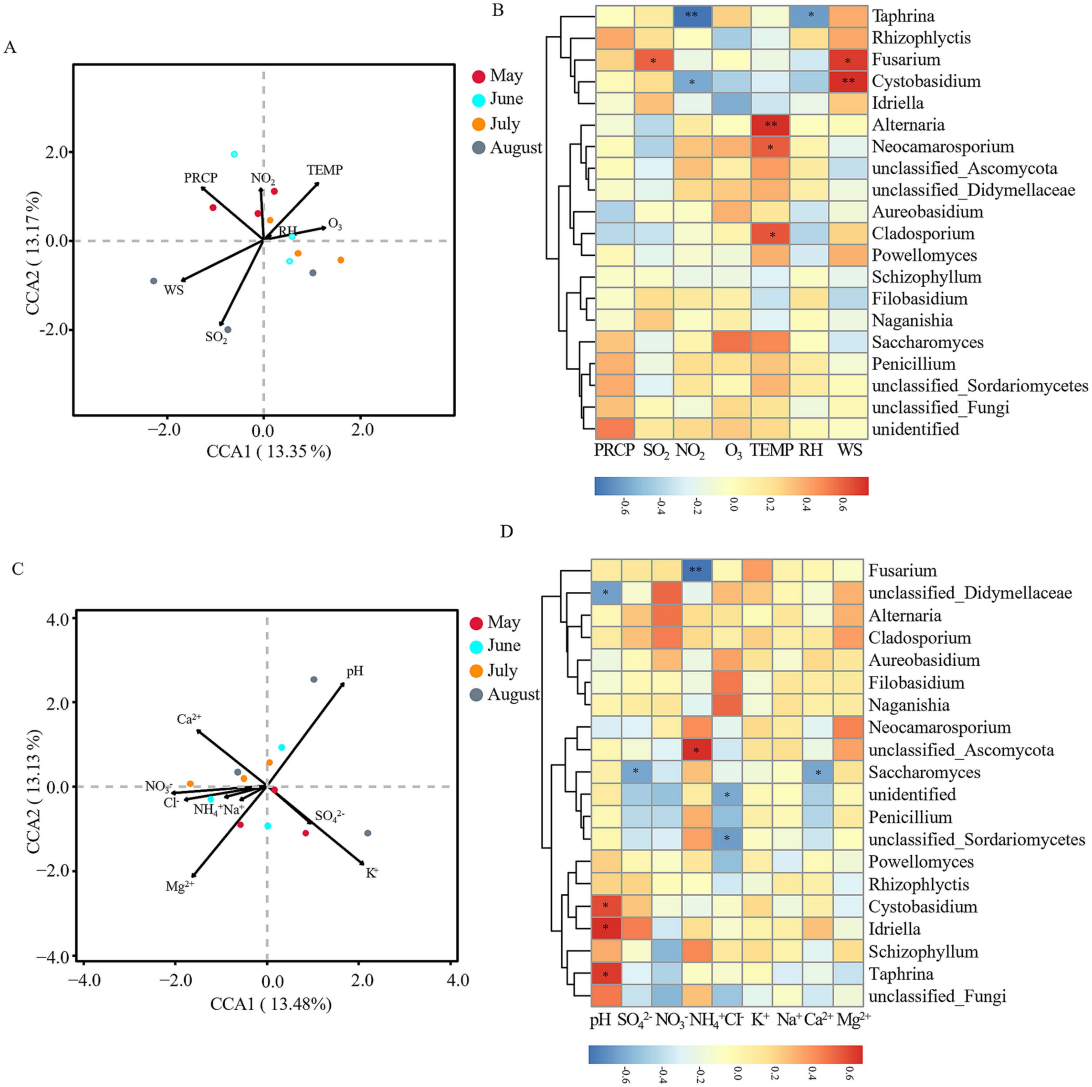
Correlation analysis between environmental factors and fungal communities. **(A)** Canonical correspondence analysis (CCA) of meteorological factors vs. fungal communities at the OTU level. **(B)** Spearman correlation analysis of meteorological factors vs. dominant fungal genera at the genus level. **(C)** CCA of precipitation physicochemical factors vs. fungal communities at the OTU level. **(D)** Spearman correlation analysis of precipitation physicochemical factors vs. dominant fungal genera at the genus level. Arrows indicate environmental variables, circles represent sample points (color-coded by month). *p < 0.05, **p < 0.01, ***p < 0.001. Meteorological factors comprised precipitation (PRCP), temperature (TEMP), relative humidity (RH), wind speed (WS), and gaseous pollutants (SO₂, NO₂, O₃). Precipitation physicochemical properties included pH and major ions (SO₄^2−^, NO₃^−^, NH₄^+^, Cl^−^, K^+^, Na^+^, Ca^2+^, Mg^2+^). Note: Color scales are automatically generated and optimized for each individual panel to best represent the structure within each dataset; therefore, direct visual comparison of color intensity between panels is not advised.

Fungal communities showed different response patterns. Meteorological factors explained 26.52% of variation, with no single factor reaching significance (Figure 7A). The Spearman heatmap revealed distinct correlations: Fusarium was positively correlated with SO_2_ (*r* = 0.594, *p* < 0.05); Taphrina (*r* = −0.774, *p* < 0.01) and Cystobasidium (*r* = −0.587, *p* < 0.05) was negatively correlated with NO_2_; Alternaria (*r* = 0.729, *p* < 0.01), Cladosporium (*r* = 0.623, *p* < 0.05), and Neocamarosporium (*r* = 0.609, *p* < 0.05) was positively correlated with TEMP; Taphrina (*r* = −0.627, *p* < 0.05) was negatively correlated with RH; and Cystobasidium (*r* = 0.734, *p* < 0.01) and Fusarium (*r* = 0.699, *p* < 0.05) was positively correlated with WS (positively, *p <* 0.05) (Figure 7B). For physicochemical factors, CCA analysis explained 26.61% of variance, with pH (*R*^2^ = 0.538, *p =* 0.032) as the only significant driver (Figure 7C). The Spearman heatmap showed: unclassified_Didymellaceae (*r* = −0.636, *p* < 0.05) was negatively correlated with precipitation pH; Idriella (*r* = 0.667, *p* < 0.05), Taphrina (*r* = 0.641, *p* < 0.05), and Cystobasidium (*r* = 0.587, *p* < 0.05) was positively correlated with pH; Saccharomyces (*r* = −0.625, *p* < 0.05) was negatively correlated with SO_4_^2−^; Fusarium (*r* = −0.797, *p* < 0.01) was negatively correlated with NH_4_^+^; unclassified_Ascomycota (*r* = 0.671, *p* < 0.05) was positively correlated with NH_4_^+^; unclassified_Sordariomycetes (*r* = −0.662, *p* < 0.05) was negatively correlated with Cl^−^; and Saccharomyces (*r* = −0.635, *p* < 0.05) was negatively correlated with Ca^2+^ (Figure 7D).

### 3.5 Microbial assembly mechanisms in atmospheric precipitation

**Figure 8:**
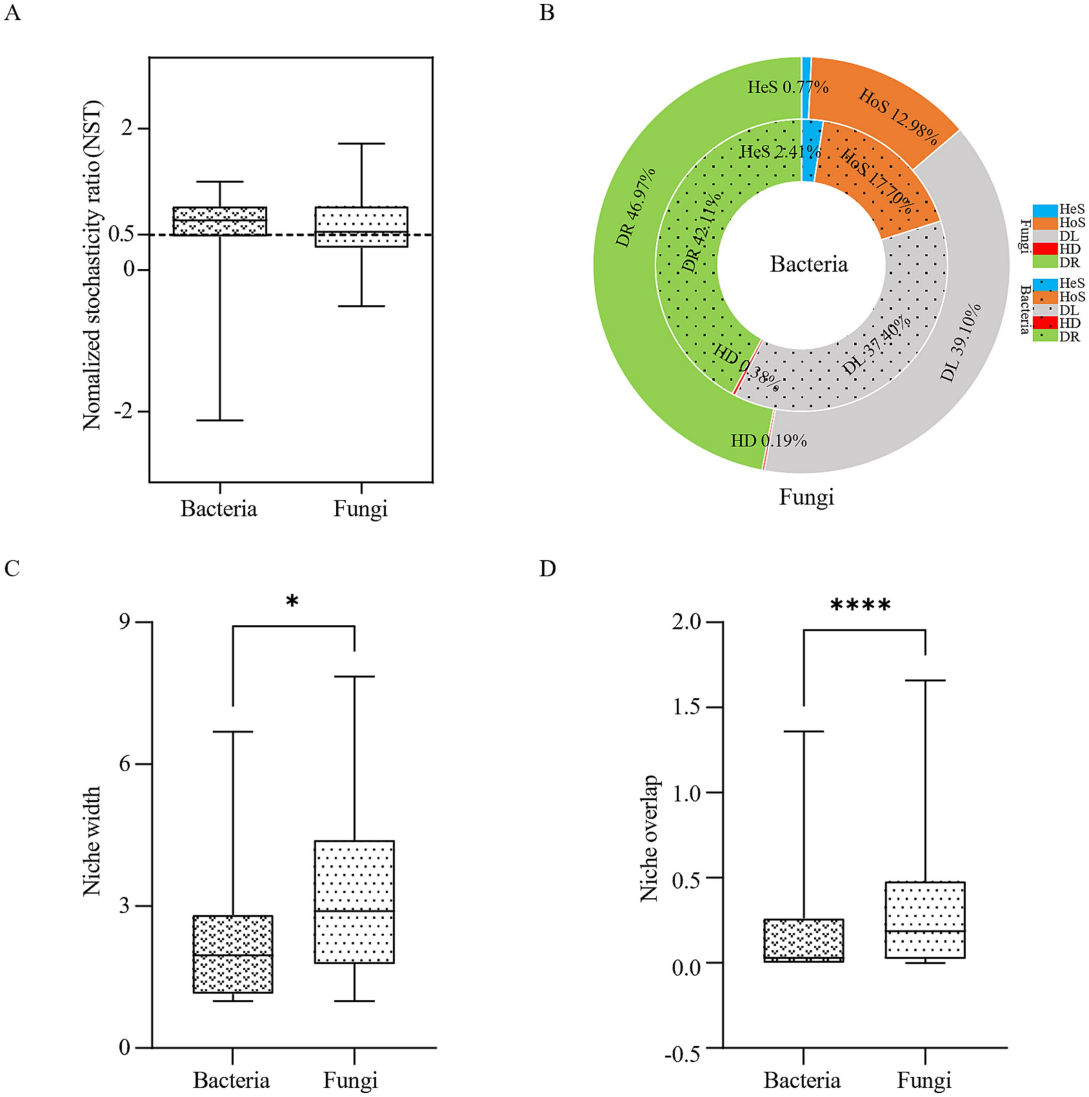
Ecological assembly processes of microbial communities in atmospheric precipitation. **(A)** Normalized stochasticity ratio (NST) analysis. **(B)** Ecological processes based on the null model (iCAMP, infer community assembly mechanisms by phylogenetic-bin-based null model analysis). HeS, Heterogeneous selection; HoS, Homogeneous selection; DL, Dispersal limitation; HD, Homogenizing dispersal; DR, Drift. **(C)** Niche width of bacteria and fungi in atmospheric precipitation. **(D)** Niche overlap of bacteria and fungi in atmospheric precipitation. *p < 0.05, **p < 0.01, ***p < 0.001, ****p < 0.0001.

NST analysis based on the “PF” null model and Bray-Curtis distance revealed that both bacterial and fungal communities exhibited mean NST values >0.5, indicating stochastic processes dominated microbial assembly (Figure 8A). Further quantification of ecological processes via iCAMP analysis revealed that bacterial community assembly is dominated by drift (DR) among stochastic processes (42.11%), followed by dispersal limitation (DL, 37.40%), while fungal community assembly is also dominated by drift (DR, 46.97%), with dispersal limitation (DL, 39.10%) as the secondary driver (Figure 8B). These results collectively demonstrate that stochasticity is a key ecological process shaping microbial communities in Baotou’s atmospheric precipitation. Boxplot analysis (Figures 8C,D) demonstrated significantly broader niche width (*p <* 0.05) and greater niche overlap (*p <* 0.0001) in fungal versus bacterial communities, suggesting fungi employ generalist strategies with intense interspecies competition/resource sharing, while bacteria occupy specialized niches to minimize competition—these complementary strategies collectively maintain community diversity and functionality.

### 3.6 Functional prediction and pathogenic potential of precipitation microbes

Functional analysis using FAPROTAX identified 28 known ecological bacterial functions for bacteria with relative abundance >0.1%. These include processes related to the carbon cycle (average relative abundance: 73.86%), nitrogen cycle (24.71%), sulfur cycle (0.16%), and pathogens (0.73%) (Table 2). The carbon cycle accounted for the highest proportion, involving 15 functions, with chemoheterotrophy (average relative abundance: 27.32%), aerobic chemoheterotrophy (12.53%), methylotrophy (13.29%), and methanol oxidation (13.27%) being the most dominant. The nitrogen cycle involved 10 functions, with ureolysis (21.93%) being the most prominent. These five functions constitute the main ecological functions of bacteria in atmospheric precipitation. Notably, the relative abundance of ureolysis in atmospheric precipitation in Baotou City was relatively high, peaking in June, which was consistent with the trend of high NH₄^+^ concentrations in precipitation (also peaking in June). This is presumably related to severe NH₄^+^ pollution in Baotou’s precipitation. The relative abundance of sulfur cycle-related functions was the lowest, with the highest value occurring in August, which aligned with the trend of high SO₄^2−^ concentrations in precipitation (peaking in August), suggesting a correlation with severe SO₄^2−^ pollution in Baotou’s precipitation.

**Table 2:** Bacterial functional groups (C/N/S cycling and pathogens) by month.

Cycle	Function	May	June	July	August
C	Chemoheterotrophy	24.88%	25.65%	27.15%	31.58%
	Aerobic chemoheterotrophy	15.86%	11.84%	4.06%	18.36%
	Fermentation	1.65%	0.55%	2.60%	0.93%
	Phototrophy	3.04%	0.51%	0.06%	1.64%
	Photoautotrophy	2.91%	0.50%	0.06%	1.15%
	Photoheterotrophy	0.13%	0.01%	0.00%	0.49%
	Hydrocarbon degradation	0.23%	0.12%	0.03%	0.45%
	Aromatic compound degradation	0.43%	0.17%	0.18%	0.84%
	Methylotrophy	7.41%	13.30%	20.54%	11.91%
	Methanol oxidation	7.35%	13.29%	20.54%	11.91%
	Cyanobacteria	2.90%	0.50%	0.06%	1.15%
	Oxygenic photoautotrophy	2.90%	0.50%	0.06%	1.15%
	Aromatic hydrocarbon degradation	0.16%	0.11%	0.03%	0.44%
	Aliphatic non-methane hydrocarbon degradation	0.16%	0.11%	0.03%	0.44%
	Dark hydrogen oxidation	0.29%	0.12%	0.01%	0.02%
	Total	70.32%	67.28%	75.41%	82.45%
N	Nitrate reduction	0.52%	1.75%	0.11%	0.61%
	Nitrate respiration	0.32%	1.40%	0.02%	0.51%
	Nitrogen respiration	0.32%	1.40%	0.02%	0.51%
	Nitrite respiration	0.28%	0.12%	0.01%	0.02%
	Ureolysis	24.29%	26.93%	22.57%	13.94%
	Nitrate denitrification	0.28%	0.12%	0.01%	0.02%
	Nitrite denitrification	0.28%	0.12%	0.01%	0.02%
	Nitrous oxide denitrification	0.28%	0.12%	0.01%	0.02%
	Denitrification	0.28%	0.12%	0.01%	0.02%
	Nitrogen fixation	0.10%	0.13%	0.72%	0.58%
	Total	26.98%	32.18%	23.46%	16.23%
S	Dark oxidation of sulfur compounds	0.01%	0.07%	0.19%	0.36%
	Total	0.01%	0.07%	0.19%	0.36%
Pathogens	Human pathogens all	0.89%	0.13%	0.16%	0.24%
	Animal parasites or symbionts	0.93%	0.14%	0.20%	0.24%
	Total	1.82%	0.27%	0.36%	0.48%
Other	Miscellaneous functions	0.87%	0.20%	0.57%	0.49%

FUNGuild analysis identified 15 known ecological fungal functional guilds with relative abundance >0.1%, dominated by pathotrophs (average relative abundance: 48.24%) and saprotrophs (48.03%), with a low abundance of symbiotrophs (3.61%) (Table 3). The most prevalent subtypes included undefined saprotrophs (40.85%; saprotrophs) and, among pathotroph guilds, fungal parasites (28.46%) and plant pathogens (15.75%), collectively representing the core ecological functions of fungi in precipitation.

**Table 3:** Fungal trophic guilds (pathotrophs/saprotrophs/symbiotrophs) by month.

Trophic mode	Guild	May	June	July	August
Pathotroph	Animal pathogens	0.71%	0.79%	0.60%	1.37%
	Fungal parasite	50.47%	18.06%	17.39%	27.93%
	Insect parasite	0.00%	1.34%	2.20%	0.00%
	Lichen parasite	0.42%	2.92%	2.45%	3.29%
	Plant pathogens	9.82%	16.44%	12.35%	24.39%
	Total	61.43%	39.55%	34.99%	56.99%
Saprotroph	Dung saprotroph	0.55%	0.45%	0.76%	0.71%
	Litter saprotroph	1.78%	0.54%	1.00%	0.06%
	Plant saprotroph	0.58%	2.12%	2.57%	2.15%
	Soil saprotroph	0.01%	0.14%	0.10%	0.27%
	Wood saprotrophs	1.72%	2.65%	8.18%	2.36%
	Undefined saprotroph	32.63%	50.83%	47.92%	32.03%
	Total	37.27%	56.74%	60.53%	37.59%
Symbiotroph	Endophyte	0.96%	3.46%	3.45%	4.02%
	Animal endosymbiont	0.23%	0.15%	0.30%	0.39%
	Arbuscular mycorrhizal fungi	0.03%	0.03%	0.42%	0.30%
	Ectomycorrhizal fungi	0.03%	0.04%	0.11%	0.50%
	Total	1.25%	3.68%	4.29%	5.21%
Other	Miscellaneous guilds	0.04%	0.03%	0.19%	0.21%

Atmospheric microorganisms, particularly potential pathogens and allergens, significantly impact human health (Jiang et al., 2022). To clarify the distribution characteristics of potential pathogenic microorganisms in the atmosphere of Baotou City, this study screened and analyzed bacterial and fungal genera associated with potential pathogens that match the “Catalog of Pathogenic Microorganisms Transmitted Among Humans” (National Health Commission of the People's Republic of China, 2023; hereinafter referred to as the “Catalog”) issued by the National Health Commission of China, with the results presented in Table 4. The results revealed that the abundance of fungal genera associated with potential pathogens in the atmosphere (0.1027–0.2420) was significantly higher than that of bacterial genera (0.0106–0.1418), indicating that fungal potential pathogens dominate the atmospheric microbial community, which is consistent with the biological characteristics of fungal spores being easily airborne and highly adaptable to the environment. Bacterial abundance peaked in June (0.1418), driven by Bacillus (0.0860) and Chryseobacterium (0.0276), likely due to favorable warm-humid conditions and increased human activity, while remaining low in other months (0.0106–0.0273). Fungal loads were highest in August (0.2420) and June (0.2378), primarily from Cladosporium (August: 0.1150), Alternaria (June: 0.0760; August: 0.0534), and Didymella (June: 0.0505), aligning with summer/autumn plant decomposition and spore release cycles. Key risks include: (1) June’s high Bacillus (containing *B. cereus*) and Pseudomonas abundances posing respiratory infection risks for immunocompromised individuals, and (2) elevated Cladosporium/Alternaria/Aspergillus levels (e.g., August Cladosporium at 0.1150) potentially exacerbating allergic respiratory diseases, especially for asthma patients.

**Table 4:** Potentially pathogenic genera by month.

Category	Genus	May	June	July	August
Bacterial genera	Bacillus	0.001349	0.086007	0.013108	0.000629
	Chryseobacterium	0.000182	0.027641	0.000000	0.000063
	Pseudomonas	0.001391	0.014688	0.000126	0.000161
	Streptococcus	0.000070	0.000384	0.006092	0.000000
	Enterococcus	0.000000	0.000210	0.004467	0.000070
	Paenibacillus	0.000161	0.005180	0.000000	0.000014
	Rhodococcus	0.001804	0.002237	0.000524	0.005026
	Nocardioides	0.001964	0.000329	0.002663	0.004775
	Kocuria	0.003677	0.005089	0.000283	0.000468
	Total bacteria	0.010598	0.141766	0.027264	0.011206
Fungal genera	Alternaria	0.015648	0.076044	0.039747	0.053429
	Cladosporium	0.032135	0.063306	0.030974	0.114981
	Fusarium	0.016891	0.006957	0.006894	0.006494
	Schizophyllum	0.007429	0.010387	0.035440	0.007333
	Didymella	0.016063	0.050471	0.019093	0.025264
	Aureobasidium	0.003979	0.017652	0.000737	0.004114
	Talaromyces	0.000183	0.001243	0.003368	0.006485
	Mortierella	0.000405	0.000723	0.004076	0.004664
	Cryptococcus	0.002207	0.002948	0.006258	0.001378
	Rhodotorula	0.001638	0.000896	0.001373	0.002804
	Curvularia	0.003584	0.001937	0.000043	0.001185
	Aspergillus	0.000202	0.000617	0.003989	0.001349
	Penicillium	0.002313	0.004644	0.015581	0.012488
	Total fungi	0.102677	0.237825	0.167572	0.241969

## 4 Discussion

As a critical scavenger of atmospheric pollutants and microbes, precipitation provides a unique window into the interactions between atmospheric chemistry and microbial ecology, particularly in industrial cities where anthropogenic emissions converge with natural dust events. Focusing on Baotou—a typical heavy industrial city in China’s arid/semi-arid region—this study reveals how chemical characteristics of precipitation shape microbial community structure, functional traits, and assembly mechanisms across monthly variations during the peak precipitation season (May–August 2023). Our findings highlight pronounced monthly shifts in microbial composition driven by industrial, agricultural, and dust-derived pollutants, underpinned by stochastic assembly processes and niche-based selection. These dynamics not only reflect the city’s mixed pollution profile but also carry implications for ecological functioning and public health.

### 4.1 Multi-source drivers of precipitation chemistry and monthly variations

Our results revealed that the precipitation in Baotou was neutral (pH = 7.04 ± 0.14) yet exhibited significantly elevated conductivity (65.38 ± 20.63 μS·cm^−1^) and total ion concentration (795.09 ± 94.68 μeq·L^−1^) compared to background and less polluted regions (Jiang et al., 2019; Zhang et al., 2014). This indicates substantial pollutant loading. The ionic composition, dominated by Ca^2+^ (27.56%), NH₄^+^ (21.57%), and SO₄^2−^ (17.35%), serves as a chemical fingerprint of Baotou’s complex “dust-agriculture-industry” pollution paradigm, consistent with its status as a heavy industrial hub prone to dust storms (Zhou et al., 2018; Gao et al., 2024).

Critically, our analysis uncovered significant monthly fluctuations in ion concentrations (Figure 1), driven by the interplay of emission sources and meteorological conditions. The peak in total ions and Ca^2+^ in June coincided with the period of most frequent dust storms, highlighting the dominance of natural dust inputs during early summer. Concurrently, the NH₄^+^ peak in June aligns with the regional spring fertilization season, suggesting a strong anthropogenic agricultural influence (Hůnová et al., 2022; Xu et al., 2020). In contrast, the highest SO₄^2−^ concentration was observed in August, likely due to intensified photochemical oxidation of SO₂ under high summer temperatures, potentially compounded by increased energy consumption (e.g., for power generation) and possibly less efficient desulfurization processes. The dilution effect of the highest precipitation amount in July effectively lowered the concentrations of most ions, demonstrating how meteorological events can transiently alter the chemical signature of precipitation. This monthly dynamics of precipitation chemistry sets the stage for the observed temporal variations in microbial communities.

### 4.2 Monthly dynamics and ecological drivers of microbial community assembly

Our study revealed a critical pattern in microbial ecology of atmospheric precipitation: while within-sample (alpha) diversity remained stable across months (Figures 2A–H), the community composition (beta diversity) underwent significant monthly restructuring (Figure 2I,J, ANOSIM bacteria: *R* = 0.586, *p* = 0.002; fungi: *R* = 0.448, *p* = 0.008). This decoupling of diversity and composition suggests that environmental filters act primarily on taxonomic identity rather than overall diversity metrics, selectively removing and favoring specific taxa while maintaining a consistent level of local richness. This pattern aligns with recent atmospheric microbiome studies and underscores the role of temporal environmental filters in reshaping microbial assemblages (Hiraoka et al., 2017; Bowers et al., 2011).

The monthly succession was driven by a dynamic interplay between dispersal events and in-situ selection pressures. The LEfSe results and community composition analysis provide mechanistic insights into this seasonal niche partitioning. The co-enrichment of bacterial families commonly found in soils (e.g., Syntrophomonadaceae, Blastocatellaceae, Myxococcaceae) in May indicates a substantial allochthonous input of soil microbes, likely dispersed via spring sandstorms (Romonosky et al., 2017). This suggests a dispersal-driven assembly process where the deposition of soil particles seeds the precipitation microbiome with taxa adapted to degrading complex organic matter (Shimkets et al., 2006).

In contrast, the August community was predominantly enriched in taxa indicative of in-situ atmospheric selection, primarily the class Acidimicrobiia and unclassified_Alphaproteobacteria. These groups are frequently linked to tolerance of UV radiation and thermal stress (Naik et al., 2022). Their dominance suggests a shift to a niche-based assembly, likely driven by summer abiotic conditions. We hypothesize that intense solar radiation promotes atmospheric photochemistry, potentially generating oxygenated organic compounds that select for taxa capable of utilizing these substrates and resisting oxidative stress (Romonosky et al., 2017). This is further supported by the significant negative correlation between O₃ and Siphonobacter (*r* = −0.764, *p* < 0.01; Figure 6B), demonstrating the inhibitory effect of photochemical products on sensitive taxa.

The transition in assembly mechanisms is also reflected in the fungal community. The replacement of Vishniacozyma (May) with Solicoccozyma (August) aligns with their documented differential tolerances to desiccation and UV radiation (Pogoda et al., 2018), and the positive correlation of Alternaria and Cladosporium with temperature (Figure 7B) links their summer peak to thermotolerance and seasonal sporulation cycles (Grinn-Gofroń et al., 2016).

### 4.3 Stochastic assembly processes and niche differentiation

Despite the clear monthly patterns driven by environmental factors, our null model analyses revealed that stochastic processes dominated the assembly of both bacterial (79.51%) and fungal (86.07%) communities (Figures 8A,B). This apparent paradox—strong monthly patterns despite stochastic dominance—can be explained by the nature of atmospheric systems. The atmosphere is an open system with high dispersal potential. Stochastic processes (drift and dispersal limitation) primarily determine which species from the regional pool arrive in a precipitation event. Subsequently, deterministic factors (e.g., O₃, pH, ion concentrations) act as a filter, shaping the relative success and abundance of those randomly arrived taxa, thus creating the observed monthly patterns. The higher stochasticity in fungi likely reflects their superior spore-mediated dispersal capabilities, making the initial arrival of species even more random (Lacey and West, 2006). The scattered distribution of July samples in the PCoA (Figures 2I,J) visually reinforces this stochasticity, likely resulting from the “scouring-mixing” effect of heavy rainfall (11.08 mm), which randomly resets the community composition.

The complementary ecological strategies of bacteria and fungi further structure the communities. Fungi displayed significantly broader niche width and greater niche overlap than bacteria (Figures 8C,D, *p* < 0.05), suggesting a “generalist” strategy where multiple fungal taxa coexist by exploiting a wide range of similar resources. Bacteria, in contrast, appeared to occupy narrower niches, minimizing competition through more specialized metabolic strategies. This divergence is a key mechanism maintaining overall community diversity and functional stability under the constraints of stochastic assembly and fluctuating environmental conditions.

### 4.4 Functional implications and health risks in an industrial city

The functional profile of the microbial communities directly reflects Baotou’s industrial environment. The high abundance of bacterial functions like methylotrophy (13.29%) and methanol oxidation (13.27%) is closely tied to industrial emissions of volatile organic compounds (e.g., methanol). Genera like *Methylobacterium_Methylorubrum* likely contribute to atmospheric “bio-purification” through methanol dehydrogenase (MDH)-mediated oxidation of these pollutants (Zhang et al., 2023). The parallel peaks of bacterial ureolysis function (21.93%) and NH₄^+^ concentration in June suggest a microbial response to alleviate ammonium toxicity through urea hydrolysis, representing a potential natural mitigation process against agricultural pollution (Mobley and Hausinger, 1989).

The health risk analysis revealed a “fungal-dominated” pattern, with significantly higher abundances of potential fungal pathogens (0.1027–0.2420) than bacterial ones. The temporal shift in risk is critical: June posed a higher bacterial risk (e.g., *Bacillus* from soil dust), while August posed a higher fungal risk (e.g., *Cladosporium*, *Alternaria*). The high abundance of these allergenic fungi in summer, driven by temperature (Figure 7B), aligns with the seasonal peak in allergic respiratory diseases (Grinn-Gofroń et al., 2016). This provides a direct link between Baotou’s industrial pollution, its modulation of microbial temporal dynamics, and public health outcomes, highlighting the need for seasonally targeted air quality advisories.

### 4.5 Implications and future research

This study, within the framework of urban atmospheric biogeochemistry, provides the first systematic insight into the microbial diversity of precipitation and its coupling with chemical characteristics in a typical industrial city of an arid/semi-arid region. It is important to note that the sample size (*n* = 12, 3 per month) was primarily constrained by the inherent scarcity of precipitation events in Baotou’s dry climate. Even during the peak precipitation season, the number of valid sampling events (i.e., those with sufficient volume, absence of prior contamination, and complete duration) is inherently limited. While this sample size is comparable to prior studies focusing on precipitation microbiology (Hiraoka et al., 2017), we acknowledge that it may limit the statistical power for detecting more nuanced relationships. Future studies would benefit from expanded sampling over multiple years to accumulate a larger dataset and to capture interannual variability. Furthermore, incorporating a spatial replication design across different functional areas of the city (e.g., industrial, residential, background sites) would greatly improve the robustness of the conclusions and allow for a more comprehensive source apportionment of both chemical and biological components.

## 5 Conclusion

In conclusion, our study demonstrates that the microbial communities in atmospheric precipitation in Baotou are shaped by a complex interplay of factors:

(1) Pollution Source Variations: The shifting dominance of dust (May–June), agricultural (June), and industrial (August) pollution sources.
(2) Meteorological Factors: Precipitation-mediated washout and dilution (July) and seasonal changes in temperature and humidity.
(3) Stochastic-Assembly-Deterministic-Filtering: Random dispersal and drift events provide the initial species pool, which is then filtered by deterministic environmental factors (e.g., O₃, SO₄^2−^, pH) to create the observed monthly compositional patterns.
(4) Ecological Strategy Differentiation: Fungi employ generalist strategies with broad niches, while bacteria occupy more specialized niches, collectively maintaining community functionality.

These findings provide a deeper mechanistic understanding of microbial ecology in the atmosphere of industrial cities. The temporal dynamics and high abundance of allergenic fungi highlight the need for seasonally targeted public health advisories, especially for vulnerable populations. Future studies integrating advanced source apportionment techniques and longer time series will further elucidate the complex interactions between specific emission sources, atmospheric conditions, and microbial bioaerosols in urban environments.

## Data Availability

The data presented in the study are deposited in the NCBI repository, accession number PRJNA1334113, https://www.ncbi.nlm.nih.gov/bioproject/PRJNA1334113.

## Footnotes

^1^ https://www.aqistudy.cn/

^2^ https://lpsn.dsmz.de/

^3^ www.biocloud.net

## References

[ref1] Bowers R. M. Clements N. Emerson J. B. Wiedinmyer C. Hannigan M. P. Fierer N. (2013). Seasonal variability in bacterial and fungal diversity of the near-surface atmosphere. Environ. Sci. Technol. 47, 12097–12106. doi: 10.1021/es402970s, PMID: 24083487

[ref2] Bowers R. M. McLetchie S. Knight R. Fierer N. (2011). Spatial variability in airborne bacterial communities across land-use types and their relationship to the bacterial communities of potential source environments. ISME J. 5, 601–612. doi: 10.1038/ismej.2010.167, PMID: 21048802 PMC3105744

[ref4] Gao Z. Wang L. Gao L. (2024). Chemical characteristics and source apportionment of atmospheric precipitation in Baotou urban area. Environ. Chem. 43, 2490–2503. doi: 10.7524/j.issn.0254-6108.2022123002

[ref5] Gardes M. Bruns T. D. (1993). ITS primers with enhanced specificity for basidiomycetes--application to the identification of mycorrhizae and rusts. Mol. Ecol. 2, 113–118. doi: 10.1111/j.1365-294x.1993.tb00005.x, PMID: 8180733

[ref6] Górka M. Pilarz A. Modelska M. Drzeniecka-Osiadacz A. Potysz A. Widory D. (2024). Urban single precipitation events: a key for characterizing sources of air contaminants and the dynamics of atmospheric chemistry exchanges. Water 16:3701. doi: 10.3390/w16243701

[ref7] Grinn-Gofroń A. Strzelczak A. Stępalska D. Myszkowska D. (2016). A 10-year study of Alternaria and Cladosporium in two polish cities (Szczecin and Cracow) and relationship with the meteorological parameters. Aerobiologia 32, 83–94. doi: 10.1007/s10453-015-9411-5, PMID: 27034536 PMC4773472

[ref8] Hiraoka S. Miyahara M. Fujii K. Machiyama A. Iwasaki W. (2017). Seasonal analysis of microbial communities in precipitation in the greater Tokyo area, Japan. Front. Microbiol. 8:1506. doi: 10.3389/fmicb.2017.01506, PMID: 28848519 PMC5554504

[ref9] Hůnová I. Brabec M. Malý M. Škáchová H. (2022). Reconstruction of daily courses of SO42−, NO3−, NH4+ concentrations in precipitation from cumulative samples. Atmos. 13:1049. doi: 10.3390/atmos13071049

[ref10] Jiang X. Q. Wang C. H. Guo J. Y. Hou J. H. Guo X. Zhang H. Y.. (2022). Global meta-analysis of airborne bacterial communities and associations with anthropogenic activities. Environ. Sci. Technol. 56, 9891–9902. doi: 10.1021/acs.est.1c07923, PMID: 35785964 PMC9301914

[ref11] Jiang B. Y. Wu Y. Li S. A. Lin T. J. He L. (2019). Chemical compositions and sources of precipitation in Shenzhen from 2010 to 2017. Environ. Chem. 38, 1872–1881. doi: 10.7524/j.issn.0254-6108.2018101004 (in Chinese)

[ref12] Lacey M. E. West J. H. (2006). The aerobiology pathway. The air Spora. Boston, MA: Springer.

[ref13] Li J. Wu H. W. Ye H. Bao Z. C. Li X. B. Zhang X. P.. (2023). Variations and source apportionment of precipitation ions in a typical acid deposition region, located in the Poyang Lake watershed: a case study of Mt. Lushan region. J. China Coal Soc. 48, 252–262. doi: 10.13225/j.cnki.jccs.2021.2075

[ref14] Lu S. Sun Y. Zhao X. Wang L. Zhen D. (2016). Impact of precipitation on Fenghe River water and aquatic microorganisms. Environ. Sci. 37, 2563–2569. doi: 10.13227/j.hjkx.2016.07.019 (in Chinese), PMID: 29964463

[ref15] Malnik V. V. Gorshkova A. S. Tomberg I. V. Netsvetaeva O. G. Molozhnikova E. V. Timoshkin O. A. (2024). Coastal water quality in Lake Baikal in Bol’shie Koty Bay, determined by the effect of atmospheric precipitation and the survival of indicator microorganisms. Water Resour. 51, 267–283. doi: 10.1134/S0097807824700787

[ref16] Ministry of Ecology and Environment of the People's Republic of China (2018). Ambient air—determination of cations (Na^+^, NH4^+^, K^+^, Mg^2+^, Ca^2+^) in precipitation—ion chromatography: HJ 1005—2018. Beijing: China Environmental Science Press.

[ref17] Mobley H. L. Hausinger R. P. (1989). Microbial ureases: significance, regulation, and molecular characterization. Microbiol. Rev. 53, 85–108. doi: 10.1128/mr.53.1.85-108.1989, PMID: 2651866 PMC372718

[ref18] Nageen Y. Wang X. Pecoraro L. (2023). Seasonal variation of airborne fungal diversity and community structure in urban outdoor environments in Tianjin, China. Front. Microbiol. 13:1043224. doi: 10.3389/fmicb.2022.1043224, PMID: 36699604 PMC9869124

[ref19] Naik A. Smithers M. Moisander P. H. (2022). Impacts of UV-C irradiation on marine biofilm community succession. Appl. Environ. Microbiol. 88:e0229821. doi: 10.1128/aem.02298-21, PMID: 34936837 PMC8863041

[ref20] National Health Commission of the People's Republic of China (2023). List of pathogenic microorganisms transmitted between humans. Beijing: National Health Commission of the People's Republic of China (in Chinese).

[ref21] Ning D. Deng Y. Tiedje J. M. Zhou J. (2019). A general framework for quantitatively assessing ecological stochasticity. Proc. Natl. Acad. Sci. USA 116, 16892–16898. doi: 10.1073/pnas.1904623116, PMID: 31391302 PMC6708315

[ref22] Pogoda C. S. Keepers K. G. Lendemer J. C. Kane N. C. Tripp E. A. (2018). Reductions in complexity of mitochondrial genomes in lichen-forming fungi shed light on genome architecture of obligate symbioses. Mol. Ecol. 27, 1155–1169. doi: 10.1111/mec.14519, PMID: 29417658

[ref23] Romonosky D. E. Li Y. Shiraiwa M. Laskin A. Laskin J. Nizkorodov S. A. (2017). Aqueous photochemistry of secondary organic aerosol of α-Pinene and α-Humulene oxidized with ozone, hydroxyl radical, and nitrate radical. J. Phys. Chem. A 121, 1298–1309. doi: 10.1021/acs.jpca.6b10900, PMID: 28099012

[ref25] Shimkets L. J. Dworkin M. Reichenbach H. (2006). "The Myxobacteria: an essay in the sociology of a genus," in The prokaryotes: volume 7: proteobacteria: delta, epsilon subclass, eds. M. Dworkin S. Falkow Rosenberg E. Schleifer K.-H. Stackebrandt E., New York, NY: Springer.

[ref26] Si L. Li Z. (2024). Atmospheric precipitation chemistry and environmental significance in major anthropogenic regions globally. Sci. Total Environ. 926:171830. doi: 10.1016/j.scitotenv.2024.171830, PMID: 38513855

[ref27] State Environmental Protection Administration of the People’s Republic of China (1992a). Determination of specific conductance in the wet precipitation: GB 13580.3—1992. Beijing: Standards Press Of China.

[ref28] State Environmental Protection Administration of the People’s Republic of China (1992b). Determination of pH value of the wet precipitationGlass electrode method: GB 13580.4—1992. Beijing: Standards Press Of China.

[ref29] State Environmental Protection Administration of the People’s Republic of China (1992c). Determination of fluoride, chloride, ntrite, nitrate, salfate in the wet precipitation-ion chromotography: GB 13580.5—1992. Beijing: Standards Press of China.

[ref30] State Environmental Protection Administration of the People’s Republic of China (1992d). Determination of ammonium in thewet precipitation: GB 13580.11—1992. Beijing: China Environmental Science Press.

[ref31] State Environmental Protection Administration of the People’s Republic of China (2004). Technical specifications for acid deposition monitoring: HJ/T 165—2004. Beijing: China Environmental Science Press.

[ref32] Takahashi S. Tomita J. Nishioka K. Hisada T. Nishijima M. (2014). Development of a prokaryotic universal primer for simultaneous analysis of Bacteria and Archaea using next-generation sequencing. PLoS One 9:e105592. doi: 10.1371/journal.pone.0105592, PMID: 25144201 PMC4140814

[ref33] Wang X. Zhang Q. Zhang Z. Li W. Liu W. Xiao N.. (2023). Decreased soil multifunctionality is associated with altered microbial network properties under precipitation reduction in a semiarid grassland. iMeta 2:e106. doi: 10.1002/imt2.106, PMID: 38868425 PMC10989785

[ref34] White T. J. Bruns T. D. Lee S. B. Taylor J. W. (1990). “Amplification and direct sequencing of fungal ribosomal RNA genes for phylogenetics,” in PCR Protocols: A Guide to Methods and Applications, eds. M. A. Innis, D. H. Gelfand, J. J. Sninsky, and T. J. White (San Diego, CA: Academic Press), 315–322.

[ref35] Xu W. Wen Z. Shang B. Dore A. J. Tang A. Xia X. P.. (2020). Precipitation chemistry and atmospheric nitrogen deposition at a rural site in Beijing, China. Atmos. Environ. 223:117253. doi: 10.1016/j.atmosenv.2019.117253

[ref37] Zhang Z. F. Wang H. L. Deli G. E. Zhu B. (2014). Chemistry characteristics of atmospheric precipitation in Waliguan. Trans. Atmos. Sci. 37, 502–508. doi: 10.13878/j.cnki.dqkxxb.20130719001 (in Chinese)

[ref38] Zhang X. Xia L. Q. Liu J. Y. Wang Z. H. Yang Y. N. Wu Y. T.. (2023). Comparative genomic analysis of a *Methylorubrum rhodesianum* MB200 isolated from biogas digesters provided new insights into the carbon metabolism of methylotrophic bacteria. Int. J. Mol. Sci. 24:7521. doi: 10.3390/ijms24087521, PMID: 37108681 PMC10138955

[ref40] Zhen S. (2012). Air quality evaluation and influence factors analysis of Baotou urban area. Baotou: School of Economics and Management Inner Mongolia University of Science and Technology.

[ref42] Zhou H. Lü C. He J. Gao M. Zhao B. Ren L.. (2018). Stoichiometry of water-soluble ions in PM2. 5: application in source apportionment for a typical industrial city in semi-arid region, Northwest China. Atmos. Res. 204, 149–160. doi: 10.1016/j.atmosres.2018.01.017

