# Supplementary figures and images for: Chemical characteristics of atmospheric precipitation and their effects on microbial diversity in Baotou, China
Source: Front Microbiol. 2025 Oct 13;16:1680819. doi: 10.3389/fmicb.2025.1680819 (PMC12554732; doi:10.3389/fmicb.2025.1680819)

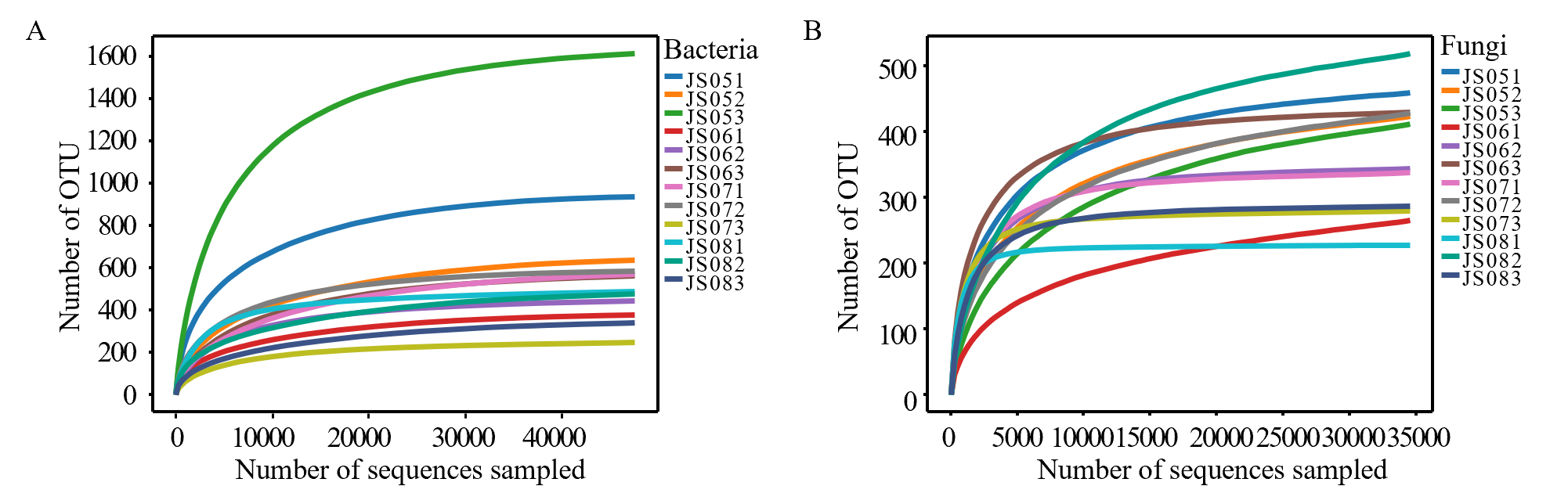

Supplement: Supplementary file 1 [file Image_1.tif]
