# Supplementary material for: Chemical characteristics of atmospheric precipitation and their effects on microbial diversity in Baotou, China
Source: Front Microbiol. 2025 Oct 13;16:1680819. doi: 10.3389/fmicb.2025.1680819 (PMC12554732; doi:10.3389/fmicb.2025.1680819)
Supplement: Supplementary file 2 [file Table_1.docx]

**Supplementary Table 1 Relative abundance of bacterial classes by month**

| class | May | June | July | August |
| --- | --- | --- | --- | --- |
| Alphaproteobacteria | 26.77% | 34.43% | 31.31% | 41.54% |
| Gammaproteobacteria | 37.94% | 25.35% | 37.79% | 16.52% |
| Bacteroidia | 10.58% | 8.17% | 9.95% | 19.22% |
| Deinococci | 3.21% | 12.06% | 4.91% | 3.27% |
| Bacilli | 1.96% | 11.48% | 8.11% | 0.88% |
| Actinobacteria | 8.54% | 5.58% | 2.10% | 4.53% |
| Cyanobacteriia | 4.41% | 0.72% | 0.21% | 0.29% |
| unclassified_Bacteria | 0.50% | 0.15% | 0.08% | 3.62% |
| Clostridia | 1.91% | 0.39% | 1.41% | 0.38% |
| Others | 4.18% | 1.66% | 4.13% | 9.74% |
| Unknown | 0.02% | 0.01% | 0.00% | 0.00% |
